# Simplified Modelling of the Edge Crush Resistance of Multi-Layered Corrugated Board: Experimental and Computational Study

**DOI:** 10.3390/ma16010458

**Published:** 2023-01-03

**Authors:** Tomasz Garbowski, Anna Knitter-Piątkowska, Piotr Winiarski

**Affiliations:** 1 Department of Biosystems Engineering, Poznan University of Life Sciences, Wojska Polskiego 50, 60-627 Poznan, Poland; 2 Institute of Structural Analysis, Poznan University of Technology, Piotrowo 5, 60-965 Poznan, Poland; 3 Schumacher Packaging Sp. z o.o., Wrocławska 66, 55-330 Krępice, Poland

**Keywords:** edge crush resistance, edge crush test, finite element analysis, analytical model, experimental data, corrugated cardboard

## Abstract

The edge crush test is the most popular laboratory test in the corrugated packaging industry. It measures the edge crush resistance of a sample in the cross-fiber direction (CD), also known as the ECT index. This parameter is widely used for the specification of the board by its producers. It is also utilized in most analytical formulas describing the load capacity of the packaging. On the other hand, the ECT value can be estimated from both analytical and numerical models based on the basic parameters of each constituent paper. Knowing the compressive strength in CD (commonly known as SCT) and the elastic properties of the individual layers, the sample geometry (i.e., the period and height of the corrugated layer), as well as the boundary conditions, the ECT value can be calculated. This is very useful as new boards can be virtually analyzed before being manufactured. In this work, both detailed numerical models based on finite elements (FE) methods and very simple analytical (engineering) models were used for the ECT calculations. All presented models were validated with experimental data. The surprising consistency and high precision of the results obtained with the simplest approach was additionally analyzed in the study.

## 1. Introduction

Corrugated cardboard plays a very important role in the economies of fast-developing countries. In a world where the social awareness of consumers related to, for example, environmental protection, a sustainable economy, and the closed product cycle is gradually growing, there is no longer any place for products that threaten or may endanger the goals of sustainable development. Earth’s resources are not inexhaustible and one should be aware of the dangers and threats associated with their careless exploitation. Producers of corrugated board and paper—although their business is based on the exploitation of one of the most important raw materials—wood obtained from logging does not affect the environment to the same extent as producers of packaging made of other, less eco-friendly materials. Since corrugated cardboard is a biodegradable material, it does not leave an imprint on the natural environment in the form of piles of hard to disposed-of material or plastic islands floating on seas and oceans. Moreover, through the use of recycled fibers in the production of paper, the factor related to deforestation is also gradually minimized. By extending the life cycle of recycled fibers in the paper production process, the amount of cut trees is reduced and, concurrently, more time is gained to rebuild forested areas.

Corrugated cardboard boxes protect their contents throughout the distribution chain and are currently used for the packaging of the vast majority of goods sold and conveyed commercially. The strength of the transport box is influenced by the weather conditions in which it is transported, resulting in the box moisture content [1,2], the method of transport itself, as well as the conditions and manner of storage [3,4,5]. The compressive strength of the box, usually measured while performing the box compression test (BCT) [6,7,8,9,10], is a key measure of the packaging resistance to mechanical stress during transport and storage.

Since the invention of corrugated cardboard and its first use in the production of packaging, engineers and scientists around the world have been looking for the appropriate methods to reliably and easily estimate the load-bearing capacity of the boxes in order to optimize the consumption of the raw material and, at the same time, adequately protect the products transported inside them. The simplest formulas, however, do not allow for a credible estimation of each type of packaging; on the other hand, more advanced methods demand specified knowledge of the designer and often also require the use of advanced numerical tools. The simplest and most frequently used method includes the McKee formula [11], which is based on the utmost popular parameters of corrugated board, that is, column crush resistance, commonly known as ECT, from the name of the test—edge crush test, and the thickness of the cardboard or bending stiffness, as well as the dimensions of the box in the base. The ECT is standardized—four methods, depending on the samples’ shapes, have been established, that is, the edge clamping method [12], the neck-down method [13], the rectangular test specimen method [13,14,15], and the edge-reinforced method [16,17]. In the literature, one can also find many modifications/adaptations aimed at increasing the effectiveness of McKee formulas provided by, for example, Frank [7], Garbowski et al. [18], Maltenfort [19], Allerby et al. [20], Schrampfer et al. [21], Kawanishi [22], and Batelka [23] and their adaptation to other, more demanding, packaging cases [8,9,10].

Nowadays, a huge emphasis is placed on ecology and optimization of the resources use (the so-called zero waste, fit-to-product, box-on-demand, etc.), therefore, the correct modeling of packaging is taking on increasing importance. The optimal design of the corrugated cardboard packages was examined by Mrówczyński et al. [24,25]. The use of recycled fibers in the production of paper and cardboard causes the fibers to become shorter and more brittle after many recovery cycles, and their ability to create a stable network of connections with other fibers keeps getting smaller over time. The utilization of starch and other agents, to halt the decline in the ability of fibers to bond to one another, causes the significant change in mechanical properties of the paper. The produced paper, although more ecological, becomes a much more heterogeneous material with a much larger number and size of initial imperfections. All geometrical and material imperfections affect the strength of paper and corrugated board; however, this is not yet a well-recognized process by engineers and scientists. Only a few works dealing with this problem can be found in the literature. Garbowski and Knitter-Piątkowska [26] took into account geometric imperfections in the analytical calculations of bending stiffness in machine direction (MD). This effect was previously investigated experimentally by Czechowski et al. [27].

Numerical techniques, based on the finite element method (FEM) [28,29] allow for accurate estimation of the load-bearing capacity of complex packaging structures [30], the determining of mechanical properties of cardboard with creases [31,32,33,34,35,36], as well as strength estimations of corrugated board packages [37,38,39,40]. FEM has also been applied to examine the torsional and transversal stiffness of orthotropic paper materials [41,42], and the bending stiffness [27,43] and buckling or post-buckling phenomena [44] of cardboard. Since the paper materials are anisotropic and the cardboard’s structure is layered, numerical simulations are demanding because the material parameters of each layer need to be known. By utilizing the method called homogenization, one can preserve the precision of the results with significant savings in computation time. In the analytical homogenization, the equations of the classical theory of strength of materials or the classical theory of laminates are used [45]. Numerical homogenization makes use of FEM, where first a numerical model of a representative volume element (RVE) is created [46]. This approach fits perfectly with cardboard issues where homogenization can be accomplished in two ways, namely homogenization to one layer or homogenization of fluting to the inner layer of the laminate. Due to its practical importance and aforementioned advantages, this procedure has been intensively developed and utilized in recent years, as reported in the literature [47,48,49,50,51,52,53,54,55,56,57,58].

FEM models can be created based on laboratory tests from which the material parameters of corrugated cardboard are obtained. Therefore, their correct definition becomes a priority. In order to comprehensively obtain all the necessary material parameters, laboratory devices can be used [59]. Through this innovative system for predicting the compressive strength of corrugated cardboard packaging, a material database is created on the basis of the bending, shear, twisting and ECT of the corrugated board samples. The BCT index is calculated for the selected project and the previously calculated material parameters of the corrugated board. However, this approach is based on research on corrugated board, which is limited only to the range of board already produced. The need to improve the corrugated board itself, driven mainly by the dynamically developing e-commerce industry, makes manufacturers outdo each other in optimizing their products by changing the composition of the produced cardboard.

Modeling from paper-to-packaging requires from the designer the knowledge of the material parameters of the constituent papers in the composition of the corrugated board. These parameters are commonly obtained by means of short-span compression tests (SCT), and by tensile tests of cardboard samples. These tests are used to determine the compressive strength and tensile strength, as well as the stiffness modulus in different directions with respect to the direction of the fibers, that is, machine direction (MD) or cross direction (CD), together with any chosen direction rotated relative to the MD by any angle. To obtain the data from the exterior surface of the specimen during the experiment, a video extensometry can be applied. This technique, comparable to digital image correlation (DIC), has been utilized by Garbowski et al. [60,61]. Such a method is distinguished by high accuracy of data capture, and is very productive in the field of experimental mechanics [42,62,63,64,65]. Correct determination of the corrugated board mechanical parameters and the knowledge of the corrugated layers geometry allows to build a model of the corrugated board. The simplest models for ECT estimation are based on empirical dependencies in which the load capacities of all papers are summed, taking into account the development factor of the fluting, namely, the so-called take-up factor. The sum of these load capacities is then scaled with the fit factor, the value of which is usually set at 0.7–0.8. In the literature, one can also find ECT models based on analytical [65,66], analytical-numerical [59,60] or experimental methods [67].

The review of several aspects of ECT testing methods, namely specimen height, test duration, and fixture-clamping effects, was drawn up by Popil [68]. It was demonstrated that the combination of the chosen testing procedures with the specific structural and strength characteristics of the cardboard being examined affects ECT values, and proved that the measurement of compression strength is sensitive to: the type of method, sample preparation, the effect of crushing, as well as the effect of test duration. In the test T 839 [12], a specimen with the dimensions 50 mm × 50 mm, irrespective of the board type, was held at its ends in the clamping fixture, which exerts pressure through its spring-loaded jaws that cover approximately two-thirds of the height of the test specimen preventing any bending of the board. According to Frank [69], T 839 may be inappropriate for some lightweight boards with relatively big calipers. Unsupported samples with waxed edges were examined in the test T 811 [16]. Their height was specifically defined for the common flute types A, B, and C. During this test, on both sides of the specimen, supporting guide blocks were positioned to ensure perpendicularity or vertical adjustment, and they were removed once the force reached the value of 22 N. Because the samples are less restrained than in the T 839 method, T 811 is expected to give lower ECT values in many cases of examining the corrugated board. Another common compression test is T 838 [13], in which the specimens with narrowed width are in the middle, thus concentrating the stress there. This technique eliminates the drawbacks of using wax to strengthen the edges in method T 811 and the consequences of exerting pressure with jaws in method T 839. In comparison to other methods, it might give the highest ECT value for lightweight boards. The influence of fluting grammage on the mechano-sorption creep of cardboard was investigated by Popil and Hojjatie [70] while using various ECT methods. Regarding the effect of sample height on ECT, the studies [68,71] have shown that ECT values decrease as specimen height increases due to the sample buckling rather than sample compression. The paper is a viscoelastic material, thus the rate of compression has an influence on ECT. The test methods indicate a compression platen running speed of 12.5 + 0.25 mm/min, however data for ECT can be received at different strain rates, and for this reason, to compare data sets, the quantification of the time relation of ECT is necessary. In general, strength properties decrease by about 7.5% per decade of strain rate change [68]. Edge crush test (ECT) measurements using the aforementioned tests on boxes crushed in varying amounts during the manufacturing process was discussed by Frank and Cash [72].

In this paper, attention is focused on the empirical ECT model based on the SCT parameters of the constituent papers of various three- and five-layer corrugated board compositions. For this purpose, SCT tests of the component papers were performed in the main directions of the material orthotropy, that is, in the MD, CD directions and in the direction rotated by 45 degrees in relation to the MD. Then, a series of ECT tests were performed on the cardboard built on these papers, loaded at different angles to the wave direction. The results of laboratory tests were used to construct an easy-to-calibrate and reliable ECT model of corrugated board. The empirical-analytical model proposed here is based on easy-to-obtain paper parameters and is much more practical than detailed models that require specific modeling knowledge, presented in our previous papers. The model is based on the theory of bearing capacity of a post-buckled plate, in which, due to the buckling of the central part, only a portion of the section is bearing the load, the so-called effective cross-section, similar to the theory used in thin-walled steel cross-sections. Due to the need to avoid laborious calculations of the critical force, the model has replaced the critical force with an empirical model that uses only the grammage of the paper (instead of its thickness) and the width of the analyzed section. The model is calibrated with only one parameter, which has a constant value for all single-walled boards and another constant value for all double-walled boards. The adopted simplification allows for an easy and very practical estimation of the ECT value based on the SCT and the grammage of the constituent papers. In addition, for comparative purposes, a numerical model of corrugated board was built on the basis of data obtained from mechanical laboratory tests of individual papers. The model was verified by laboratory tests of corrugated cardboard, and then the influence of various types and sizes of imperfections on the results of numerical analyses was checked. The observations clearly show that both material and geometric imperfections have a significant impact on the edge crush resistance of the corrugated board.

## 2. Materials and Methods

### 2.1. Laboratory Tests

**Figure 1:**
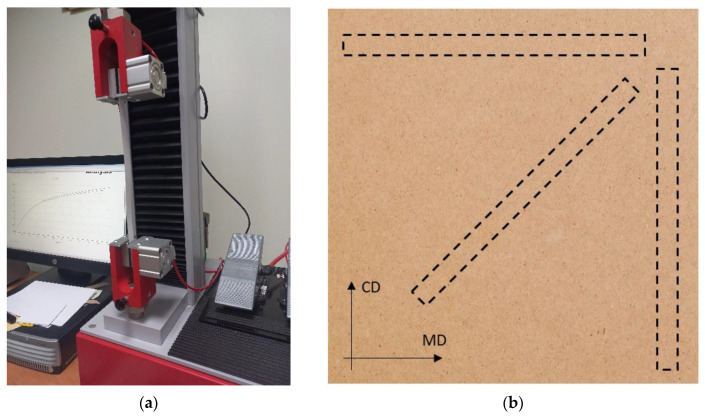
Paper tensile stiffness test: (**a**) laboratory device; (**b**) samples cut in the directions: MD, CD and 45 degrees.

In order to build the correct analytical or numerical model of the corrugated board, it was necessary to perform a series of laboratory tests of all component papers and obtain the necessary material parameters. Aiming to correctly define the computational models, the papers were first subjected to tensile tests (see Figure 1a) in three main directions, namely MD (machine direction), CD (cross direction), and at 45 degrees with respect to MD (see Figure 1b), and short-span compression tests (SCT) in the CD (i.e., direction transverse to the direction of the fibers). To find the tensile parameters of paper samples, a Testometric laboratory device was used in accordance with the ISO 9073-3 standard [73].

**Figure 2:**
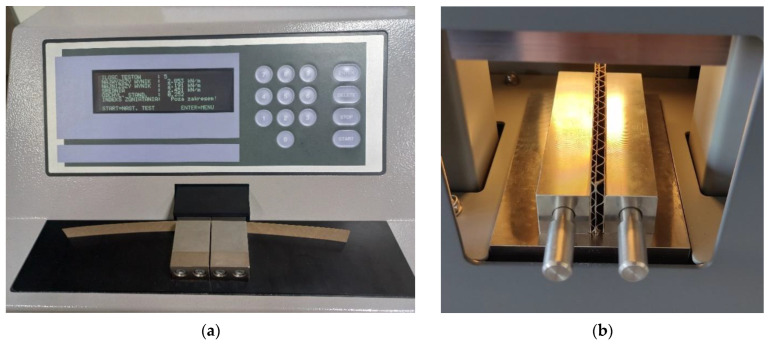
Laboratory testing machines: (**a**) short-span compression tester; (**b**) edge crush tester.

The parameters of the paper in compression were identified only in one direction, that is, in the CD, in which the corrugated board usually works. For this purpose, a Short Span Compression Tester from TMI, model 17–36 (Messmer Büchel-Industrial Physics, LLC, Veenendaal, The Netherlands), was used in accordance with the ISO 9895 standard [74]. The SCT device is shown in Figure 2a.

The reference values of the edge crush test (ECT) for various corrugated board samples were measured according to the PN-EN ISO 3037:2013-12 [15] standard. In such a test, the sample is 100 mm long and 25 mm high. The ECT tests were performed on the device from FEMAT (Poznań, Poland), model ECT-10-21 (see Figure 2b). As can be seen in Figure 2b, the samples were each time supported by metal blocks that prevented the samples from buckling or tilting during the test.

It is worth mentioning that in corrugated cardboard, all papers are arranged in such a way that the direction of the fibers coincides with the direction of the wave. This means that in the ECT test, all layers are loaded in the CD. Therefore, in the presented procedure, the compressive strength of papers was tested only in the CD direction. The tensile tests were only necessary to determine the orthogonal stiffnesses in the linear-elastic orthotropic model.

All tests, both paper and board tests, were carried out under standard laboratory conditions, that is, 22 °C and 50% humidity. In all laboratory tests, eight material samples were prepared (samples of paper cut in selected directions and samples of corrugated board). As the tests were performed in accredited laboratories, the statistical analysis of the obtained results was omitted in this paper and, therefore, all presented results are the mean values.

### 2.2. Corrugated Board

In order to develop a correct computational model of corrugated board in the ECT test, a representative group of different cardboards was selected. In total, six popular compositions of three-layer and five-layer corrugated board were chosen for the tests, the main parameters of which are listed in Table 1.

Table 2 compiles the grammage values of the tested cardboards and the measured cross-sectional heights. In order to systematize the presented results, the cardboard ID was adopted, consisting of a symbol describing the type of wave and the basis weight of individual corrugated cardboard samples.

The corrugated boards presented in Table 2 are widely used for the production of packaging and, at the same time, constitute a wide spectrum of its different types, from single-walled with a height of almost 1.6 mm to double-walled with a height of over 6.7 mm.

### 2.3. Component Papers

All paper samples were examined in two main orthotropic directions, that is, in the MD and CD, and at an angle of 45 degrees to the MD. Markings and grammages of individual component papers for all cardboard samples are listed in Table 3.

### 2.4. Numerical Model

In order to model the ECT test correctly and comprehensively, the finite element method was used. On the basis of the measured stiffness and compressive and tensile strength in the two main orthotropic directions, as well as in the direction rotated by 45 degrees, the remaining material parameters were determined. The modulus of shear stiffness in the plane was determined from the following formula:

(1) $$\frac{1}{E_{x\alpha }}=\frac{\cos^{2}\left(\alpha \right)}{E_{x}}+\left(\frac{1}{G_{xy}}-\frac{2\nu_{xy}}{E_{x}}\right)\sin^{2}\left(\alpha \right)\cos^{2}\left(\alpha \right)+\frac{\sin^{4}\left(\alpha \right)}{E_{y}}.$$

Because in the tests carried out, the examinations were performed in the direction of 45 degrees relative to the *x* direction, and the direction *x* = MD and *y* = CD, Formula (1) therefore takes the form:

(2) $$G_{xy}={\left(\frac{4}{E_{45}}-\frac{1}{E_{x}}-\frac{1}{E_{y}}+\frac{2\nu_{xy}}{E_{x}}\;\right)}^{-1}.$$

In the above formulas, $E_{x}$ is the stiffness modulus in the MD direction, $E_{y}$ is the stiffness modulus in the CD direction, $E_{45}$ is the stiffness in the direction rotated 45 degrees from the MD direction, and $\nu_{xy}$ is the Poisson’s ratio in the xy plane, which can be determined by the empirical formula proposed by Baum [21]:

(3) $$\nu_{xy}=0.293\sqrt{\frac{E_{y}}{E_{x}}}.$$

Due to the special case of loading the corrugated board sample at the edges along the CD direction, which is observed in the ECT test, only the SCT parameter in the CD direction was used to model the inelastic behavior. As a simplification, it is assumed that in the ECT test, the compressive strength of the paper in other directions is not activated, nor is the tensile strength. Therefore, the constitutive model consists of the orthotropic linear elastic part and the nonlinear isotropic inelastic part. As was already indicated, the numerical model was built while applying the finite element (FE) method. A shell element with four nodes and four Gaussian points with an approximate dimension of one side of the element of about 0.35 mm was used for the analysis. Calculations were made in the ABAQUS FEA 2021 commercial software.

**Figure 3:**
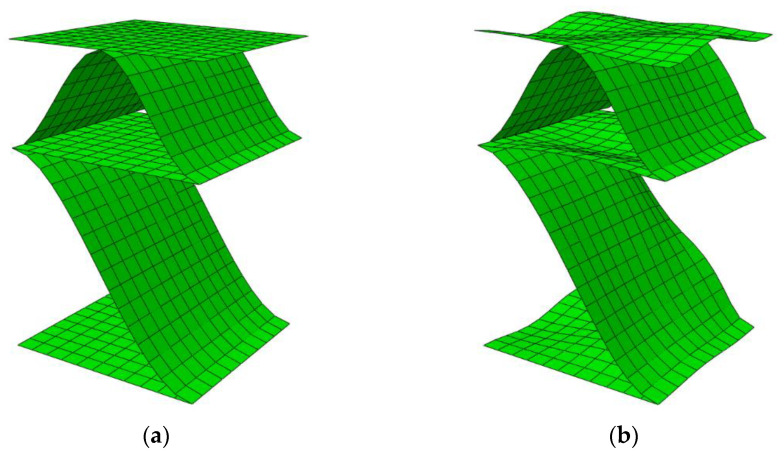
Model of BC-790 five-layered corrugated board: (**a**) without imperfections; (**b**) with imperfections (scale ×10).

In the numerical analysis of thin shell elements that can undergo large displacements or rotations, apart from material non-linearity, geometric non-linearity should also be taken into account. This allows for correct modelling when thin layers of paper start to buckle. Unfortunately, numerical models without initial imperfections are often characterized by a lack of convergence, thus geometric or material imperfections should be taken into account in the modeling. In order to account for imperfections in the FE model of the ECT sample, the geometrical imperfections were introduced by changing the position of nodes in the model, so that the shape of the geometric imperfection corresponded to the lowest eigenvectors buckling model (see Figure 3).

The numerical model shown in Figure 3 is only a small part of a whole model and is intended to show the shape of buckling mode applied to account for imperfections. The full model covers the entire sample, that is, the model with dimensions of 100 mm × 25 mm. All geometric features of the numerical models are presented in Table 1. The numerical model itself, the description of the boundary conditions, and the loading condition and convergence studies were carried out and shown in our previous work [60,61].

### 2.5. Empirical Model

In the definition of the simplest empirical ECT model of corrugated board, the SCT (in CD) of the component papers and the take-up factor of corrugated layers are used. The model can be defined as follows:

(4) $$ECT=A\overset{n}{\underset{i=1}{\sum }}SCT_{i}\cdot \alpha_{i}.$$

In this model, the SCT values of individual layers are summed up and multiplied by the take-up factor, which for flat layers equals 1. The fitting factor A is the only parameter that is used to correlate the result obtained with the model and the actual result from the laboratory test. It is very impractical, because in this way we adjust the test results for both single and double-wall corrugated board with one parameter only, which appears to be different for almost any cardboard configuration.

### 2.6. Analytical-Empirical Model

**Figure 4:**
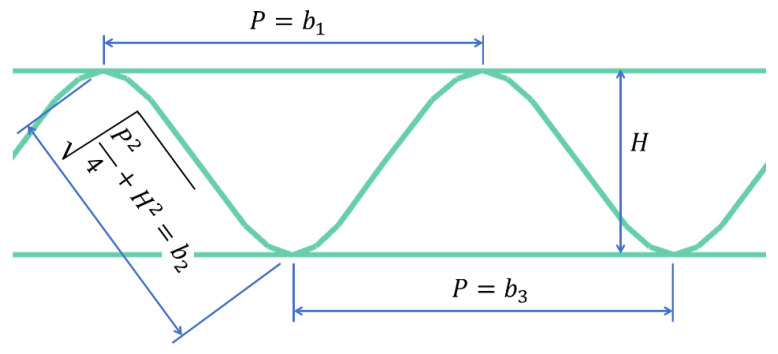
Cross-section of three-layer corrugated cardboard.

In order to better represent the actual behavior of a cardboard sample, where imperfections often occur, an analytical–empirical model can be used. It is assumed that the model takes into account both the SCT parameter, which describes the compressive strength of individual paper segments (both liners between the tops of the waves and curvilinear segments of fluting) and the critical force, which describes the initiation of buckling. It does not require the correlation of the empirical parameter A that appears in Equation (4). Such a model can be written, for example, in the following form:

(5) $$ECT=\overset{n}{\underset{i=1}{\sum }}SCT_{i}\cdot \alpha_{i}\cdot \gamma_{i},$$

where the $\gamma_{i}$ parameter is a factor reducing the static load capacity on compression of individual layers. This coefficient takes into account the buckling of the layers and depends on the geometry of the flute and the grammage of the given component paper. It would be more correct to base this parameter on paper thickness, but this parameter is rarely determined in the laboratory and does not appear in paper specifications supplied by paper mills. The formula describing the parameter $\gamma_{i}$ can be written as:

(6) $$\gamma_{i}=SCT_{i}^{-1/2}{\left(\frac{H}{a}\frac{g_{i}}{b_{i}}\right)}^{1/2}\leq 1,$$

where a is a parameter that should be selected once and for all on the basis of experimental observations (empirical adjustment), H is the height of the cross-section, $g_{i}$ is the basis weight of a given layer, $b_{i}$ is the width of individual segments in the cross-section of corrugated board (details in Figure 4).

**Figure 5:**
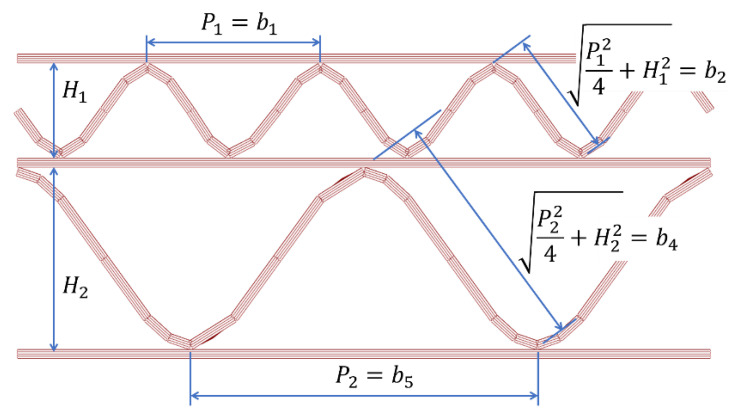
Cross-section of five-layer corrugated cardboard.

In the case of corrugated board with a double wall, a better solution is to omit the parameter H from the Equation (6), which then takes the form:

(7) $$\gamma_{i}=SCT_{i}^{-1/2}{\left(\frac{1}{a^{*}}\frac{g_{i}}{b_{i}}\right)}^{1/2}\leq 1,$$

where $a^{*}$ is again a parameter to be determined on the basis of experimental observations, $g_{i}$ is the basis weight of a given paper, while $b_{i}$ is the width of individual segments in the cross-section of corrugated board (details in Figure 5). The value of the width $b_{3}$ (inner flat layer) was assumed a priori as 0.7 of the smaller of the two wavelengths, that is:

(8) $$b_{3}=\min\left(P_{1},P_{2}\right)\times 0.7,$$

which, in this case, leads to: $b_{3}=0.7P_{1}$.

It is worth noting that the parameter $\gamma_{i}$ is the square root of the ratio between the critical force and the compressive strength of the selected corrugated board segment. Instead of tedious analytical [75,76] or numerical [77,78] calculations of the critical load of the analyzed segment, its empirical form is assumed here. This factor somewhat reduces the width of each segment that actually carries the load during the edge crush test.

## 3. Results

### 3.1. Laboratory Tests Results

Table 4 summarizes the results of the SCT in CD and tensile stiffness tests in three directions (MD, CD and 45 degrees) obtained for all constituent papers of the six selected corrugated boards: (a) B-410; (b) C-590; (c) E-480; (d) BC-790; (e) BE-600; (f) BE-590. Each paper was tested 3–4 times, and when the results were widely dispersed, additional tests were performed. Average values are presented in the table.

### 3.2. The ECT Estimations by Numerical and Analytical-Empirical and Empirical Models

Table 5 presents the empirically determined coefficients presented in Equations (4), (6) and (7). These coefficients were determined using least squares minimization. It is worth noting that the coefficient A was selected individually for each quality of corrugated board and then averaged (columns 3 and 4 in Table 5). The coefficients a and $a^{*}$ were computed individually for single-walled and double-walled cardboards.

The simplest empirical model described by Equation (4) uses the coefficient A, which can be selected for each cardboard quality separately (column 2 of Table 5), and it may also be selected separately for each type of corrugated board (column 3 of Table 5) or commonly for all cardboards (column 4 of Table 5).

Finally, five models listed below were used for ECT estimation:

- Model 1: proposed analytical–empirical model, Equation (5);
- Model 2: numerical model FEM1 with imperfection 2%;
- Model 3: numerical model FEM2 with imperfection 9%;
- Model 4: empirical model described by Equation (4) with coefficient from Table 5 (column 3);
- Model 5: empirical model described by Equation (4) with coefficient from Table 5 (column 4).

Table 6 presents the estimation results using the proposed Model 1 (see Equation (5)) and two numerical models—Model 2 and Model 3—which differ only in the assumed scale of imperfections.

Table 7 summarizes the estimation error obtained while using different models. The abbreviations M1 to M5 denote the model numbers that have already been described.

**Figure 6:**
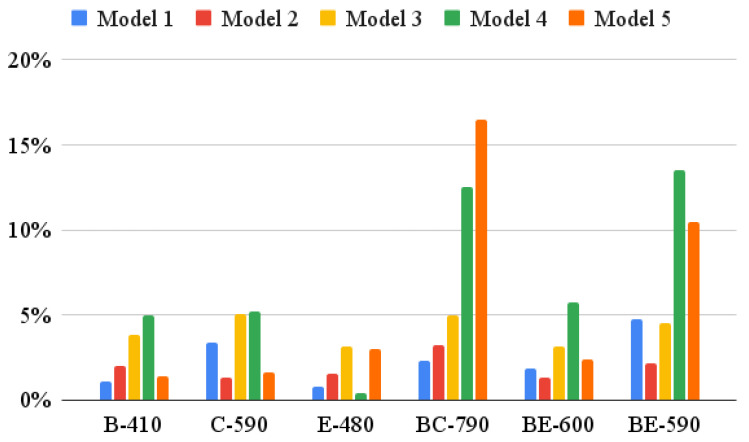
Absolute estimation error obtained using five different models.

Figure 6 shows the absolute estimation errors obtained while utilizing different models. It is worth noting that the estimations obtained using the models based on Equation (4) clearly deviate from the estimations which make use of the other models.

## 4. Discussion

The test results presented in Table 4 made it possible to build a material model, which was then used in a numerical model based on the finite element method for ECT estimation. Both numerical models used for ECT calculations differ only in the size of imperfections that must be included in the model to obtain correct results. This is due to the fact that in the real sample, imperfections are built in a priori, therefore numerical models should also have initial geometrical imperfections. The problem is that it is not known a priori where exactly and how big these imperfections are. One way to incorporate the initial imperfections is to determine the first buckling mode and scale the obtained displacements to obtain a new geometry that takes these displacements into account. Here, two different magnitudes of these displacements have been chosen: 2% and 9%.

As can be seen in Table 6 (columns 4–5) and Table 7 (columns 3–4), the estimation results obtained while using numerical model M2 are more correct than those obtained using the model M3. In the M2 model, imperfections of 2% of the displacement values obtained from the buckling analysis for the first lowest mode were used. In the M3 model, imperfections at the level of 9% of the value of displacements on each wall of the corrugated board cross-section were applied. Unfortunately, the actual imperfections are not known, however, the obtained estimations show that they amount to about 3–5% of the buckling displacements. This is due to the fact that the values obtained using the M2 model were higher than the reference values in each analyzed case, while the M3 model underestimated the ECT value in each case.

Models M4 and M5 based on Equation (4), although quite effective in the case of single-walled cardboard, were completely ineffective in the case of double-walled corrugated board. This is shown very clearly by the results presented in Table 7 and Figure 6. Even the determination of the correlation coefficient A separately for two types of board did not help—the stimulation error for double-walled board still oscillated between +/−13%. This is, of course, related to the very simple form of the model (see Equation (4)).

By contrast, model M1 proposed here behaved very correctly in each case of the analyzed corrugated board. The results obtained using the M1 model are burdened with an estimation error not exceeding +/−5%. The numerical models M2 and M3 are characterized by similar accuracy. For the correct construction of the M1 model, the following ingredients are necessary: (a) the compressive strength of the constituent papers (SCT), (b) their grammage and (c) the geometrical characteristics of the corrugated layers. Correct numerical modeling requires additionally: (d) paper stiffness in each orthotropic direction (i.e., in MD, CD and 45 deg) and, of course, (e) specialized software, as well as (f) necessary expert knowledge in computer modeling. Therefore, the proposed analytical–empirical model allows to significantly speed up the estimation of ECT without losing the accuracy of the obtained results compared to numerical models. In addition, this model is much more precise than the currently used simplified models.

## 5. Conclusions

The paper presented an analytical–empirical model for the estimation of corrugated board resistance to edge crushing. On the basis of the obtained results, also comparing the accuracy of the estimation with currently applicable laboratory models and with advanced numerical models based on the finite element method, it is concluded that the model is: (1) as precise as numerical models; (2) much simpler to use than numerical models; (3) much more precise than simplified models; and (4) it requires only a few readily available parameters. As shown in the work on six different grades of corrugated board, simplified modeling provides a very practical tool that, in addition to being accurate, is also very easy to calibrate because the model is based on commonly known and easily obtained paper and corrugated board parameters. The common use of the proposed here model in the laboratory practice of companies producing corrugated board will not only significantly improve the process of selecting the appropriate inputs, but also through high precision, achieve significant savings of the raw material.

## Figures and Tables

**Table 1:** Component papers and geometry of the corrugated boards.

Wave	Grammage	Component	Height	Period	Take-Up
Type	(g/m^2^)	Papers	(mm)	(mm)	Factor
B	410	TL3125/WS120/TL3125	2.55	6.34	1.337
C	590	KLB170/S.C.175/KLB170	3.63	7.95	1.427
E	480	TLWC160/WS 135/TLW160	1.16	3.50	1.236
BC	790	KLB170/WS135/WS80/WS135/KLB170	-	-	-
BE	600	TLW140/WS95/WS80/WS95/TL3125	-	-	-
BE	590	TL3125/WS95/WS80/WS95/TL3125	-	-	-

**Table 2:** Basic parameters of cardboards.

Wave Type	Grammage (g/m^2^)	Cardboard ID	Height (mm)
B	410	B-410	2.912
C	590	C-590	4.110
E	480	E-480	1.586
BC	790	BC-790	6.740
BE	600	BE-600	4.150
BE	590	BE-590	4.120

**Table 3:** Designation of the constituent papers of the tested corrugated board.

Cardboard ID	Type of Paper	Grammage (g/m^2^)	Layer
B-410	TL3	125	Liner ext.
	WS	120	Fluting
	TL3	125	Liner intern.
C-590	KLB	170	Liner ext.
	S.C.	175	Fluting
	KLB	170	Liner intern.
E-480	TLWC	160	Liner ext.
	WS	135	Fluting
	TLW	160	Liner intern.
BC-790	KLB	170	Liner ext.
	WS	135	Fluting (B)
	WS	80	Liner
	WS	135	Fluting (C)
	KLB	170	Liner intern.
BE-600	TLW	140	Liner ext.
	WB	95	Fluting (E)
	WB	80	Liner
	WB	95	Fluting (B)
	TL3	125	Liner intern.
BE-590	TL3	125	Liner ext.
	WS	95	Fluting (E)
	WS	80	Liner
	WS	95	Fluting (B)
	TL3	125	Liner intern.

**Table 4:** Average SCT values in CD and average tensile stiffness values of all constituent papers in the MD, CD and in 45 degree directions.

Cardboard ID	Paper ID	SCT-CD (N/mm)	TS-CD (N/mm)	TS-MD (N/mm)	TS-45 (N/mm)
B-410	TL3-125	2.14	373.33	1012.7	572.68
	WS-120	2.09	365.06	1024.6	516.50
	TL3-125	2.09	381.15	1058.3	595.08
C-590	KLB-170	3.28	527.77	1472.1	929.12
	SC-175	4.18	686.08	1476.1	924.73
	KLB-170	3.19	568.05	1445.1	956.22
E-480	TLWC-160	2.75	412.14	1043.6	635.01
	WS-135	2.13	365.02	1067.5	533.52
	TLW-160	2.43	443.86	1102.1	667.03
BC-790	KLB-170	3.39	618.88	1534.2	990.02
	WS-135	2.19	369.08	1113.5	572.41
	WS-80	1.50	317.09	699.14	445.02
	WS-135	2.23	385.91	1147.4	623.54
	KLB-170	3.30	592.74	1418.9	838.15
BE-600	TLW-140	2.61	505.95	999.95	622.60
	WS-95	1.69	331.94	872.70	498.63
	WS-80	1.42	273.16	812.83	424.82
	WS-95	1.52	290.66	885.54	508.44
	TL3-125	2.13	440.63	1082.2	623.26
BE-590	TL3-120	2.26	412.94	961.28	586.98
	WS-95	1.50	294.34	756.40	427.40
	WS-80	1.47	343.54	696.51	459.84
	WS-95	1.75	332.01	854.73	474.40
	TL3-125	2.32	413.77	883.13	588.29

**Table 5:** Empirically determined coefficients A (column 2–4), a (column 5) and $a^{*}$ (column 6).

Cardboard ID	A Equation (4)			a Equation (6)	$a^{*}$ Equation (7)
(1)	(2)	(3)	(4)	(5)	(6)
B-410	0.781	0.791	0.819	52	-
C-590	0.778				
E-480	0.815				
BC-790	0.728	0.848	0.819	-	18
BE-600	0.869				
BE-590	0.947				

**Table 6:** Reference (measured) ECT values and estimation values with empirical–analytical and numerical models.

Cardboard ID	ECT (ref) (N/mm)	ECT (M1) (N/mm)	ECT (M2) (N/mm)	ECT (M3) (N/mm)
B-410	5.48	5.54	5.59	5.27
C-590	9.68	9.35	9.81	9.19
E-480	6.37	6.32	6.47	6.17
BC-790	10.41	10.65	10.75	9.89
BE-600	8.95	9.12	9.07	8.67
BE-590	9.68	9.22	9.89	9.24

**Table 7:** Estimation error obtained using five different models.

Cardboard ID	Error (M1) (%)	Error (M2) (%)	Error (M3) (%)	Error (M4) (%)	Error (M5) (%)
B-410	1.09	2.01	−3.83	4.98	1.39
C-590	−3.41	1.34	−5.06	5.21	1.61
E-480	−0.78	1.57	−3.14	0.45	−2.99
BC-790	2.31	3.27	−5.00	12.51	16,49
BE-600	1.90	1.34	−3.13	−5.76	−2.43
BE-590	−4.75	2.17	−4.55	−13.54	−10.48

## Data Availability

Not applicable.

## References

[1] Stott R.A. (2017). Compression and stacking strength of corrugated fibreboard containers. Appita Technol. Innov. Manuf. Environ..

[2] Junli W., Quancheng Z. (2006). Effect of moisture content of corrugated box on mechanical properties. J. Lanzhou Jiaotong Univ..

[3] Zhang Y.-L., Chen J., Wu Y., Sun J. (2011). Analysis of hazard factors of the use of corrugated carton in packaging low-temperature yogurt during logistics. Procedia Environ. Sci..

[4] Gallo J., Cortés F., Alberdi E., Goti A. (2021). Mechanical behavior modeling of containers and octabins made of corrugated cardboard subjected to vertical stacking loads. Materials.

[5] Whitsitt W.J., Gander J.W., McKee R.C. (1968). Effect of Box Dimensions and Combined Board Creep Life on Box Creep Life.

[6] Urbanik T.J., Frank B. (2006). Box compression analysis of world-wide data spanning 46 years. Wood Fiber Sci..

[7] Frank B. (2013). Corrugated Box Compression—A Literature Survey. Packag. Technol. Sci..

[8] Garbowski T., Gajewski T., Grabski J.K. (2021). Estimation of the compressive strength of corrugated cardboard boxes with various perforations. Energies.

[9] Garbowski T., Gajewski T., Grabski J.K. (2021). Estimation of the compressive strength of corrugated cardboard boxes with various openings. Energies.

[10] Mrówczyński D., Garbowski T., Knitter-Piątkowska A. (2021). Estimation of the compressive strength of corrugated cardboard boxes with shifted creases on the flaps. Materials.

[11] McKee R.C., Gander J.W., Wachuta J.R. (1963). Compression strength formula for corrugated boxes. Paperboard Packag..

[12] (2009). Edge Compression Test for Strength of Corrugated Fiberboard Using the Clamp Method (Short Column Test).

[13] (2009). Edge Crush Test Using Neckdown.

[14] (1997). Edgewise Crush Resistance of Corrugated Fiberboard.

[15] (2013). Corrugated Fibreboard—Determination of Edgewise Crush Resistance (Unwaxed Edge Method).

[16] (2009). Edgewise Compressive Strength of Corrugated Fibreboard (Short Column Test).

[17] (2002). Corrugated Fibreboard—Determination of Edgewise Crush Resistance—Waxed Edge Method.

[18] Garbowski T., Niziałek-Łukawska M., Kuca M. (2019). Analytical verification of popular McKee’s formula. Pol. Pap. Rev..

[19] Maltenfort G. (1956). Compression strength of corrugated containers. Fibre Contain..

[20] Allerby I.M., Laing G.N., Cardwell R.D. (1985). Compressive strength—From components to corrugated containers. Appita Conf. Notes.

[21] Schrampfer K.E., Whitsitt W.J., Baum G.A. (1987). Combined Board Edge Crush (ECT) Technology.

[22] Kawanishi K. (1989). Estimation of the compression strength of corrugated fibreboard boxes and its application to box design using a personal computer. Packag. Technol. Sci..

[23] Batelka J.J., Smith C.N. (1993). Package Compression Model.

[24] Mrówczyński D., Knitter-Piątkowska A., Garbowski T. (2022). Non-Local Sensitivity Analysis and Numerical Homogenization in Optimal Design of Single-Wall Corrugated Board Packaging. Materials.

[25] Mrówczyński D., Knitter-Piątkowska A., Garbowski T. (2022). Optimal Design of Double-Walled Corrugated Board Packaging. Materials.

[26] Garbowski T., Knitter-Piątkowska A. (2022). Analytical determination of the bending stiffness of a five-layer corrugated cardboard with imperfections. Materials.

[27] Czechowski L., Kmita-Fudalej G., Szewczyk W., Gralewski J., Bieńkowska M. (2021). Numerical and experimental study of five-layer non-symmetrical paperboard panel stiffness. Materials.

[28] Zienkiewicz O.C., Taylor R.L., Zhu J.Z. (2013). The Finite Element Method: Its Basis and Fundamentals.

[29] De Borst R., Crisfield M.A., Remmers J.J.C., Verhoosel C.V. (2012). Nonlinear Finite Element Analysis of Solids and Structures.

[30] Fadiji T., Coetzee C.J., Berry T.M., Ambaw A., Opara U.L. (2018). The efficacy of finite element analysis (FEA) as a design tool for food packaging: A review. Biosyst. Eng..

[31] Domaneschi M., Perego U., Borgqvist E., Borsari R. (2017). An industry-oriented strategy for the finite element simulation of paperboard creasing and folding. Packag. Technol. Sci..

[32] Awais M., Tanninen P., Leppänen T., Matthews S., Sorvari J., Varis J., Backfol K. (2018). A computational and experimental analysis of crease behavior in press forming process. Procedia Manuf..

[33] Thakkar B.K., Gooren L.G.J., Peerlings R.H.J., Geers M.G.D. (2008). Experimental and numerical investigation of creasing in corrugated paperboard. Philos. Mag..

[34] Beex L.A.A., Peerlings R.H.J. (2009). An experimental and computational study of laminated paperboard creasing and folding. Int. J. Solids Struct..

[35] Giampieri A., Perego U., Borsari R. (2011). A constitutive model for the mechanical response of the folding of creased paperboard. Int. J. Solids Struct..

[36] Leminen V., Tanninen P., Pesonen A., Varis J. (2019). Effect of mechanical perforation on the press-forming process of paperboard. Procedia Manuf..

[37] Garbowski T., Jarmuszczak M. (2014). Numerical strength estimate of corrugated board packages. Part 1. Theoretical assumptions in numerical modeling of paperboard packages. Pol. Pap. Rev..

[38] Garbowski T., Jarmuszczak M. (2014). Numerical strength estimate of corrugated board packages. Part 2. Experimental tests and numerical analysis of paperboard packages. Pol. Pap. Rev..

[39] Park J., Chang S., Jung H.M. (2020). Numerical prediction of equivalent mechanical properties of corrugated paperboard by 3D finite element analysis. Appl. Sci..

[40] Park J., Park M., Choi D.S., Jung H.M., Hwang S.W. (2020). Finite element-based simulation for edgewise compression behavior of corrugated paperboard for packing of agricultural products. Appl. Sci..

[41] Nordstrand T., Carlsson L. (1997). Evaluation of transverse shear stiffness of structural core sandwich plates. Compos. Struct..

[42] Słonina M., Dziurka D., Smardzewski J. (2020). Experimental research and numerical analysis of the elastic properties of paper cell cores before and after impregnation. Materials.

[43] Jamsari M.A., Kueh C., Gray-Stuart E.M., Dahm K., Bronlund J.E. (2019). Experimental and numerical performance of corrugated fibreboard at different orientations under four-point bending test. Packag. Technol. Sci..

[44] Urbanik T.J., Saliklis E.P. (2003). Finite element corroboration of buckling phenomena observed in corrugated boxes. Wood Fiber Sci..

[45] Allaoui S., Benzeggagh M.L., Aboura Z., Talbi N. (2004). Elastic behaviour of corrugated cardboard: Experiments and modeling. Compos. Struct..

[46] Biancolini M.E. (2005). Evaluation of equivalent stiffness properties of corrugated board. Compos. Struct..

[47] Garbowski T., Jarmuszczak M. (2014). Homogenization of corrugated paperboard. Part 1. Analytical homogenization. Pol. Pap. Rev..

[48] Garbowski T., Jarmuszczak M. (2014). Homogenization of corrugated paperboard. Part 2. Numerical homogenization. Pol. Pap. Rev..

[49] Garbowski T., Gajewski T. (2021). Determination of transverse shear stiffness of sandwich panels with a corrugated core by numerical homogenization. Materials.

[50] Garbowski T., Knitter-Piątkowska A., Mrówczyński D. (2021). Numerical homogenization of multi-layered corrugated cardboard with creasing or perforation. Materials.

[51] Mrówczyński D., Knitter-Piątkowska A., Garbowski T. (2022). Numerical Homogenization of Single-Walled Corrugated Board with Imperfections. Appl. Sci..

[52] Ramírez-Torres A., Penta R., Rodríguez-Ramos R., Merodio J., Sabina F.J., Bravo-Castillero J., Guinovart-Díaz R., Preziosi L., Grillo A. (2018). Three scales asymptotic homogenization and its application to layered hierarchical hard tissues. Int. J. Solids Struct..

[53] Ramírez-Torres A., Di Stefano S., Grillo A., Rodríguez-Ramos R., Merodio J., Penta R. (2018). An asymptotic homogenization approach to the microstructural evolution of heterogeneous media. Int. J. Non-Linear Mech..

[54] Hohe J. (2003). A direct homogenization approach for determination of the stiffness matrix for microheterogeneous plates with application to sandwich panels. Compos. Part B Eng..

[55] Buannic N., Cartraud P., Quesnel T. (2003). Homogenization of corrugated core sandwich panels. Compos. Struct..

[56] Abbès B., Guo Y.Q. (2010). Analytic homogenization for torsion of orthotropic sandwich plates. Compos. Struct..

[57] Marin G., Srinivasa P., Nygårds M., Östlund S. (2021). Experimental and finite element simulated box compression tests on paperboard packages at different moisture levels. Packag. Technol. Sci..

[58] Suarez B., Muneta M.L.M., Sanz-Bobi J.D., Romero G. (2021). Application of homogenization approaches to the numerical analysis of seating made of multi-wall corrugated cardboard. Compos. Struct..

[59] FEMat BSE Systems. http://fematsystems.pl/bse-system_en.

[60] Garbowski T., Grabski J.K., Marek A. (2021). Full-field measurements in the edge crush test of a corrugated board—Analytical and numerical predictive models. Materials.

[61] Garbowski T., Knitter-Piątkowska A., Marek A. (2021). New edge crush test configuration enhanced with full-field strain measurements. Materials.

[62] Hägglund R., Åslund P.E., Carlsson L.A., Isaksson P. (2010). Measuring thickness changes of edgewise compression loaded corrugated board panels using digital image correlation. J. Sandw. Struct. Mater..

[63] Viguié J., Dumont P.J.J. (2013). Analytical post-buckling model of corrugated board panels using digital image correlation measurements. Comp. Struct..

[64] Fadiji T., Coetzee C.J., Opara U.L. (2020). Evaluating the displacement field of paperboard packages subjected to compression loading using digital image correlation (DIC). Food Bioprod. Process..

[65] Maier G., Bolzon G., Buljak V., Garbowski T., Miller B., Kuczma M., Wilmanski K. (2010). Synergic combinations of computational methods and experiments for structural diagnoses. Computer Methods in Mechanics.

[66] Garbowski T., Imbierowicz R. (2014). Sensitivity analysis of edge crush test. Przegląd Pap..

[67] Dimitrov K., Heydenrych M. (2009). Relationship between the edgewise compression strength of corrugated board and the compression strength of liner and fluting medium papers. South. For..

[68] Popil R.E. (2012). Overview of recent studies at IPST on corrugated board edge compression strength: Testing methods and effects of interflute buckling. BioResources.

[69] Frank B. (2007). Revisiting clamped ECT. Corrugat. Int..

[70] Popil R.E., Hojjatie B. (2010). Effects of component properties and orientation on corrugated container endurance. Packag. Technol. Sci..

[71] Wilson C.J., Frank B. (2009). An evaluation of ECT sample height for small flute board grades and Box Manufacturer’s Certification compliance. Tappi J..

[72] Frank B., Cash D. (2022). Edge crush testing methods and box compression modeling. Tappi J..

[73] (1989). Textiles—Test methods for nonwovens—Part 3: Determination of tensile strength and elongation.

[74] (2008). Paper and board—Compressive strength—Short-span test.

[75] Patel P., Nordstrand T., Carlsson L.A. (1997). Local buckling and collapse of corrugated board under biaxial stress. Compos. Struct..

[76] Nyman U., Gustafsson P.J. (2000). Material and structural failure criterion of corrugated board facings. Compos. Struct..

[77] Daxner T., Flatscher T., Rammerstorfer F.G. Optimum Design of Corrugated Board under Buckling Constraints. Proceedings of the 7th World Congress on Structural and Multidisciplinary Optimization.

[78] Ma Y., Gong Z., Liang Zhao L., Han Y., Wang Y. (2014). Finite Element Analysis of Buckling of Corrugated Fiberboard. Open Mech. Eng. J..

